# Single‐nucleotide substitution T to A in the polypyrimidine stretch at the splice acceptor site of intron 9 causes exon 10 skipping in the *ACAT1* gene

**DOI:** 10.1002/mgg3.275

**Published:** 2017-02-08

**Authors:** Hideo Sasai, Yuka Aoyama, Hiroki Otsuka, Elsayed Abdelkreem, Mina Nakama, Tomohiro Hori, Hidenori Ohnishi, Lesley Turner, Toshiyuki Fukao

**Affiliations:** ^1^ Department of PediatricsGraduate School of MedicineGifu UniversityGifuJapan; ^2^ Department of Biomedical SciencesCollege of Life and Health SciencesChubu UniversityKasugaiJapan; ^3^ Department of PediatricsFaculty of MedicineSohag UniversitySohagEgypt; ^4^ Division of Clinical GeneticsGifu University HospitalGifuJapan; ^5^ Discipline of GeneticsMemorial University of NewfoundlandSt John'sNFCanada

**Keywords:** *ACAT1*, exon skipping, mitochondrial acetoacetyl‐CoA thiolase deficiency, polypyrimidine stretch, splice acceptor site, T2 deficiency

## Abstract

**Background:**

β‐ketothiolase (T2, gene symbol *ACAT1*) deficiency is an autosomal recessive disorder, affecting isoleucine and ketone body metabolism. We encountered a patient (GK03) with T2 deficiency whose T2 mRNA level was <10% of the control, but in whom a previous routine cDNA analysis had failed to find any mutations. Genomic PCR‐direct sequencing showed homozygosity for c.941‐9T>A in the polypyrimidine stretch at the splice acceptor site of intron 9 of *ACAT1*. Initially, we regarded this variant as not being disease‐causing by a method of predicting the effect of splicing using in silico tools. However, based on other findings of exon 10 splicing, we eventually hypothesized that this mutation causes exon 10 skipping.

**Methods:**

cDNA analysis was performed using GK03's fibroblasts treated with/without cycloheximide (CHX), since exon 10 skipping caused a frameshift and nonsense‐mediated mRNA decay (NMD). Minigene splicing experiment was done to confirm aberrant splicing.

**Results:**

cDNA analysis using fibroblasts cultured with cycloheximide indeed showed the occurrence of exon 10 skipping. A minigene splicing experiment clearly showed that the c.941‐9T>A mutant resulted in transcripts with exon 10 skipping. There are few reports describing that single‐nucleotide substitutions in polypyrimidine stretches of splice acceptor sites cause aberrant splicing.

**Conclusion:**

We showed that c.941‐9T>A induces aberrant splicing in the *ACAT1* gene. Our ability to predict the effects of mutations on splicing using in silico tools is still limited. cDNA analysis and minigene splicing experiments remain useful alternatives to reveal splice defects.

## Introduction

β‐Ketothiolase deficiency (OMIM #203750, *607809), a defect of mitochondrial acetoacetyl‐CoA thiolase (T2, EC 2.3.1.9, gene symbol *ACAT1*, RefSeq NM_000019.3), is an autosomal recessive disorder, affecting isoleucine and ketone body metabolism (Fukao et al. 2014). Since 1971 (Daum et al. 1971), more than 100 T2‐deficient patients have been identified (Mitchell and Fukao 2001). Human T2 cDNA is about 1.5 kb long and encodes a precursor protein of 427 amino acids, including a 33‐amino‐acid leader polypeptide (Fukao et al. 1990). *ACAT1* spans approximately 27 kb and contains 12 exons (Kano et al. 1991). We have identified more than 70 gene mutations, 15% of which cause aberrant splicing (Fukao et al. 2010). Most are located at the highly conserved dinucleotide “ag” at the splice acceptor site and “gt” at the splice donor site. We also identified some exonic mutations that cause aberrant splicing by activating cryptic splice sites within their exons (Nakamura et al. 2001; Fukao et al. 2008) or by altering the consensus sequences of exonic splice enhancer sites (Fukao et al. 2010).

It is still a challenge to predict whether single‐nucleotide substitutions in polypyrimidine stretches of splice acceptor sites cause disease. We herein describe that a c.941‐9T>A mutation in the polypyrimidine stretch at the splice acceptor site of intron 9 of *ACAT1* induces exon 10 skipping and causes β‐ketothiolase deficiency.

## Materials and Methods

### Case presentations

#### GK03

A case report on GK03 was previously published (Middleton et al. 1986). Briefly, he was a Laotian male infant. When he was 10 months old, he was admitted to a medical center because of vomiting, diarrhea, and acidosis. Three of the patient's siblings had died of unknown causes at <6 months of age. The results of a neurological examination were grossly normal. Laboratory findings showed severe ketoacidosis; the arterial blood pH was 7.16 and the serum concentration of bicarbonate was 6 mEq/L. The urine test for ketones was strongly positive. The patient was treated with intravenous fluids and bicarbonate, and the acidosis resolved in 24 h. Urinary organic acid analysis revealed increased excretion of 3‐hydroxybutyrate and 2M3HB, but not tiglylglycine. Potassium ion‐activated acetoacetyl‐CoA thiolase activity was reduced in the fibroblasts of this patient; the +K^+^/−K^+^ ratio was reported to be 1.1. Northern blot analysis showed a decreased amount (<10% of that in the control) of T2 mRNA (Fukao et al. 1990) and routine cDNA analysis did not reveal any mutations at that time. The molecular basis of T2 deficiency in this patient was not resolved. Growth and development were normal.

#### GK124

GK124 was a girl born to American parents with no significant family history. She had previously been well with normal development and health. At 5 years and 11 months of age, she had a 4‐day history of fever, lethargy, and decreased appetite. She had also experienced one episode of emesis. The night prior to admission, her parents had noted that she was breathing quite heavily and had a reduced level of consciousness. She was also described as being delusional. When she was admitted to hospital, she had extreme acidosis. Capillary blood gas revealed pH of 7.058, bicarbonate 3.4 mmol/L, and base excess −26 mmol/L; her lactate was 2.4 mmol/L, glucose was 4.8 mmol/L, sodium 138 mmol/L, potassium 4.1 mmol/L, and chloride 110 mmol/L. The anion gap was 21 mmol/L. Initial urine had 3+ ketones. The acylcarnitine profile was normal and plasma carnitine levels were slightly low (these were collected 3 days after admission). Initial urine organic acids collected on the day of admission showed large amounts of lactic acid, 3‐hydroxybutyrate, and acetoacetate. Urine organic acid analyses were repeated several times. On several occasions, 2M3HB was present along with 3‐hydroxybutyrate and acetoacetate.

She was treated with intravenous fluid including glucose and bicarbonate. Her blood gas normalized in 48 h. Cranial CT scan was normal. She was discharged after 10 days. After discharge, it was noted that her speech was slightly slurred and her gait was abnormal. Cranial magnetic resonance imaging (MRI) revealed bilateral small ovoid areas with an increased T2 signal within the basal ganglia at the junction of the posterior putamen and globus pallidus.

Although metabolic investigations were not suggestive of any specific metabolic condition, a diagnosis of Τ2 deficiency was suspected because of severe metabolic acidosis with a sequela of a basal ganglia lesion. Acetoacetyl‐CoA thiolase activity in fibroblasts of 12 nmol/min/mg protein was within the normal range (8.9–20.6 nmol/min/mg protein), but the enzyme was not stimulated by the addition of potassium to the reaction. Mutation analysis of *ACAT1* reported no disease‐associated mutations in exons 1–12. The results of targeted array CGH with exon‐level resolution were normal (both mutation analysis and array CGH were performed at GeneDx).

Although metabolic, enzymatic, and molecular studies did not confirm a diagnosis of deficiency, the suspicion of this diagnosis led to repeat molecular testing. This testing was performed at the Baylor College of Medicine, 3 years after the first molecular testing. The results revealed homozygosity for c.941‐9T>A in intron 9.

The corresponding author was consulted about the pathological meaning of c.941‐9T>A. At the time of writing, this patient is 11 years of age and is doing well academically. However, she continues to have an abnormal gait with dystonic posturing of the left leg. Her most recent MRI performed at 11 years of age showed stable bilateral basal ganglion changes. She has not suffered any further episodes of acidosis.

### Ethical consideration

This study was approved by the Ethics Committee of the Graduate School of Medicine, Gifu University, Gifu, Japan, and was carried out in accordance with the principles of the Declaration of Helsinki.

### Fibroblasts and enzyme assay

Fibroblasts from GK03 and controls were cultured in Eagle's minimal essential medium containing 10% fetal calf serum. Acetoacetyl‐CoA thiolase enzyme assay was performed as described previously (Williamson et al. 1971) with modifications (Fukao et al. 1996). We calculated the mean values and standard errors of acetoacetyl‐CoA thiolase activity, in the absence and presence of potassium ions, of three independent experiments.

### Immunoblot analysis

Immunoblot analysis was performed using fibroblasts’ protein extract, as described previously (Fukao et al. 1997), with a mixture of an anti‐human T2 and anti‐SCOT (succinyl‐CoA:3‐ketoacid CoA transferase) antiserum used as the first antibody.

### Mutation screening at the genomic level

Genomic DNA was purified using Sepa Gene kits (EIDIA, Tokyo, Japan) from GK03's fibroblasts. Mutation analysis was performed by PCR amplification of each exon and its boundaries (at least 18 bases from the exon/intron boundaries in both directions) with a pair of intronic primers described previously (Fukao et al. 1998), followed by direct sequencing. The genomic sequence was obtained from RefSeq (accession number NG_009888.1/NM_000019.3).

### Nonsense‐mediated mRNA decay (NMD) inhibition

To determine whether any observed reduction of the transcript was due to NMD (Maquat 2005), we inhibited NMD in fibroblasts using CHX (Sigma, St. Louis, MO, USA), a general protein translation inhibitor. Fibroblasts were left untreated or cultured in the presence of 200 μg/mL CHX for 7 h before RNA extraction. This analysis was performed as described previously (Hernan et al. 2011).

### cDNA analysis

Total RNA was isolated from fibroblasts using ISOGEN kits (Nippon Gene, Tokyo, Japan). Total RNA (5 μg) was reverse‐transcribed in 20 μL of 50 mm Tris–HCl pH 7.5, 75 mm KCl, 3 mm MgCl_2_, 10 mm dithiothreitol, 0.5 mm dNTPs, and 200 U M‐MLV reverse transcriptase (Life Technologies, Rockville, MD, USA) with a primer mixture including 5 pmol of each of T2‐specific antisense primer (T2 135 5′‐^c.1360^TGACCCACAGTAGTCACAC‐3′), *GAPDH*‐specific antisense primer (*GAPDH*3 5′‐^c.1040^GTGCTCTTGCTGGGGCTG‐3′), and oligo dT primers. The above preparation was incubated at 37°C for 1 h. One microliter of this cDNA solution served as a PCR template. Sequencing was performed after the full coding sequence of T2 cDNA had been amplified (c. 1–1284).

For cDNA analysis with/without CHX treatment, the cDNA fragment c.732–c.1142 was amplified using the following primer set:

Primer T2 Ex8 (sense) 5′‐^c.732^TCAACCAGATGTAGTGGT‐3′.

Primer T2 114 (antisense) 5′‐^c.1142^ACAGCTCCTCCATTGATATTC‐3′.

To evaluate the total amount of mRNA products, *GAPDH* cDNA was also amplified with the following primer set:

*GAPDH*1 (sense) 5′‐^c.4^GGGAAGGTGAAGGTC‐3′.

*GAPDH*2 (antisense) 5′‐^c.1015^AGGGGTCTTACTCCTTGGAG‐3′.

These cDNA sequences were obtained from RefSeq (accession number NM_002046.4 for *GAPDH*, and NM_000019.3 for T2). After 30 PCR cycles, amplified fragments were separated by electrophoresis on a 5% (w/v) polyacrylamide gel and a 1% (w/v) agarose gel, and extracted using a Geneclean II kit (BIO 101, Vista, CA, USA). Sequences of the amplified fragments were confirmed by direct sequencing.

### Quantitative real‐time PCR

Quantitative real‐time PCR was performed using a LightCycler FastStart DNA Master CYBR Green I Kit (Roche, Mannheim, Germany). The amplification reaction was performed using a LightCycler 1.5 (Roche). Specific primers were designed for the target gene using the Primer 3 software program (http://bioinfo.ut.ee/primer3-0.4.0/primer3/). To amplify wild‐type T2 cDNA, primers for exon 9 and exon 10 were used. *GAPDH* cDNA was amplified as an internal control.

*ACAT1*‐9L (sense) 5′‐^c.814^CAGAAAGAAAATGGCACAGTAACAG‐3′.

*ACAT1*‐10R (antisense) 5′‐^c.976^GAAAATCAATAGGTTCTACAGCAGC‐3′.

*GAPDH* L (sense) 5′‐^c.930^CATTTCCTGGTATGACAACG‐3′.

*GAPDH* R (antisense) 5′‐^c.1047^TCCTCTTGTGCTCTTGCTGG‐3′.

The amplification reaction for the SYBR Green I assays contained 1× SYBR Green I PCR Mastermix and 250 nm of each primer. The PCR conditions were 95°C for 10 min, followed by 50 cycles of 95°C for 10 sec, 63°C for 15 sec, and 72°C for 1 min. Melting curves were generated after amplification. Ct values of T2 and *GAPDH* cDNAs for each sample were determined by the second derivative maximum method. Then, the amount of T2 cDNA of GK03 fibroblasts relative to that of control fibroblasts was calculated with adjustment using the relative *GAPDH* cDNA amount.

### Minigene splicing experiment

A wild‐type exon 10 minigene splicing construct was previously established using a pCAGGS eukaryote expression vector (Fukao et al. 2013). To make a mutant construct, in vitro mutagenesis was performed using the QuikChange Site‐Directed Mutagenesis Kit (Stratagene, La Jolla, CA, USA). Two micrograms of these expression vectors were transfected into 5 × 10^5^ SV40‐transformed fibroblasts using Lipofectamine 2000 (Invitrogen, Carlsbad, CA, USA). At 48 h after transfection, RNA was extracted from the cells. The first‐strand cDNA was transcribed with a rabbit β‐globin‐specific antisense primer (β‐glo2) (5′‐^c.461^AGCCACCACCTTCTGATA‐3′) and then amplified with the Ex10 (*Eco*RI) primer on T2 exon 10, and another rabbit‐specific antisense primer (β‐glo3) (5′‐^c.443^GGCAGCCTGCACCTGAGGAGT‐3′) to amplify the chimeric cDNA of human T2 and rabbit β‐globin. A study of normal and aberrant splicing constructs was performed by sequencing and 5% polyacrylamide gel electrophoresis of the PCR‐amplified cDNA fragments.

## Results and Discussion

**Figure 1:**
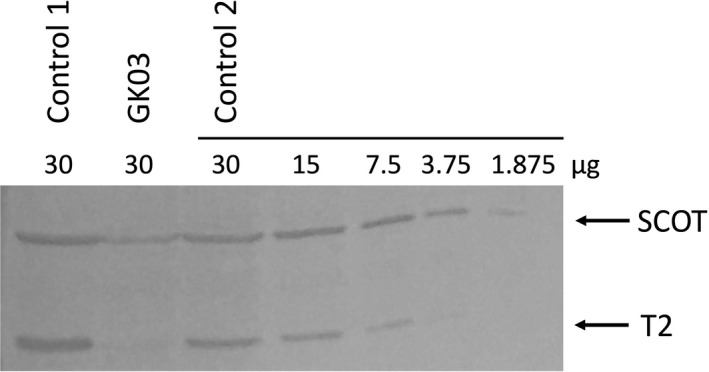
Immunoblot analysis. The amount of fibroblast protein extract applied is indicated in each lane. T2 protein was obtained from GK03 fibroblasts and two healthy control fibroblasts. The first antibody was a mixture of an anti‐T2 antibody and an anti‐SCOT (succinyl‐CoA: 3‐ketoacid CoA transferase) antibody. The positions of the bands for T2 and SCOT are indicated by arrows.

We investigated the molecular basis of T2 deficiency in a patient, GK03, whose fibroblasts had a reduced T2 mRNA level of <10% that of controls by Northern blot analysis (Fukao et al. 1990). Previous routine cDNA analysis had failed to find any mutations. In immunoblot analysis (Fig. 1), no T2 protein was detected in GK03's fibroblasts when 30 μg of protein extract was applied. In our system, the detection limit of T2 protein was at 3.75 μg of protein extract in twofold serial dilution samples of control fibroblasts. Hence, the level of T2 protein in GK03 fibroblasts was estimated to be less than one‐eighth the control level. Genomic PCR and direct sequencing identified a homozygous substitution of c.941‐9T>A with no other mutations. This intronic variant resulted in a slight decrease in the Senapathy and Shapiro score (S&S score) from 90.48 to 85.17. Therefore, we initially regarded it as a non‐disease‐causing substitution. We hypothesized that another mutation in GK03 produced a reduced amount of wild‐type T2 mRNA. However, we failed to discover any mutations in the promoter region or the 3′ noncoding regions including the polyadenylation site that could affect mRNA stability.

We recently identified another T2‐deficient patient (GK124) who had the same c.941‐9T>A substitution with no other mutations. We also recently showed, using minigene splicing experiments, that mutations (c.949G>A, c.951C>T) within the exonic splicing enhancer sequence (ESE, SF2/ASF) within exon 10 resulted in exon 10 skipping (Fukao et al. 2010; Otsuka et al. 2016). Moreover, such effects of ESE mutations were canceled by a substitution from C to G at the first nucleotide of exon 10. This implies that the intron 9 splice acceptor site is weak and the ESE within exon 10 is necessary for accurate splicing, although the S&S score of this acceptor site is high. Consequently, even if the c.941‐9T>A variant results in a subtle change in the S&S score, we hypothesized that this variant also caused exon 10 skipping.

**Figure 2:**
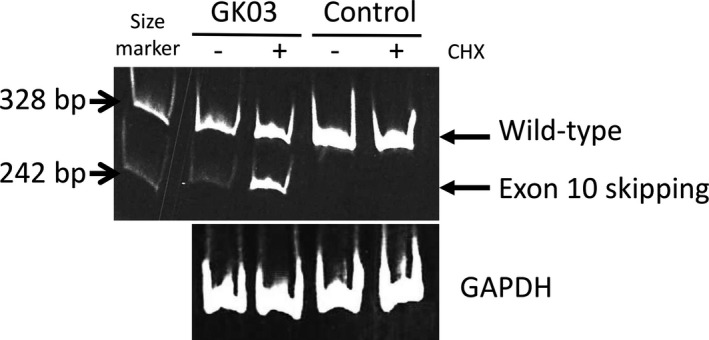
cDNA analysis using CHX‐treated fibroblasts. Five percent polyacrylamide gel electrophoresis of amplified T2 cDNA fragments using RNAs extracted from cycloheximide (CHX)‐treated and ‐untreated fibroblasts from the patient and a control. Bands corresponding to normal transcripts and those with exon 10 skipping are depicted by arrows.

First, we performed cDNA analysis again using GK03's fibroblasts treated with/without cycloheximide (CHX), since exon 10 skipping caused a frameshift and nonsense‐mediated mRNA decay (NMD). As shown in Fig. 2, without CHX, cDNA with the normal length was predominant. A faint band with a shorter length was also visible in polyacrylamide gel electrophoresis, but it was hardly detectable in agarose gel electrophoresis. This explains why we only detected the wild‐type cDNA sequence in the previous study. Real‐time PCR showed that the wild‐type T2 mRNA level decreased to 8% (±1%) of that in the control (data not shown); this value was compatible with the findings of a previous Northern blot analysis. In contrast, upon treatment with CHX, cDNA with a shorter length due to exon 10 skipping was clearly identified in GK03's fibroblasts. This indicates that transcripts with exon 10 skipping undergo NMD. The band with exon 10 skipping was not identified in control fibroblasts, even with CHX treatment. Accordingly, we confirmed that exon 10 skipping had occurred in GK03's fibroblasts.

**Figure 3:**
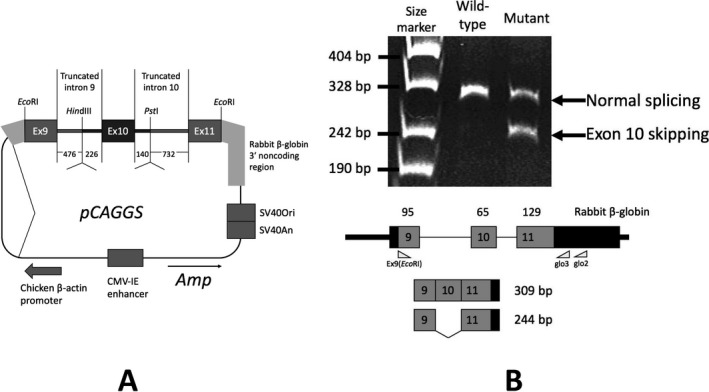
Minigene splicing experiment. (A) Schematic presentation of minigene splicing construct. pCAGGS expression vector was used for this construct. The minigene construct had a T2 gene fragment from c.842 of exon 9 and intron 9 (from +1 to a *Hin*dIII site, 476 bp) and intron 10 (from a *Pst*I site to −1, 732 bp), and exon 11 (to c. 1122). In the cases of mutant constructs, the region around exon 10, highlighted in black, was replaced as a cassette. (B) Minigene splicing experiment. Detection of chimeric cDNAs derived from transfected minigenes. First‐strand cDNA was reverse‐transcribed using the glo2 primer. cDNA amplification was performed using Ex9 (*Eco* RI) and glo3 primers. Those fragments were electrophoresed on 5% polyacrylamide gel. Normal splicing and exon 10 skipping produced 309‐bp and 244‐bp PCR fragments, respectively.

Next, we examined whether c.941‐9T>A can induce exon 10 skipping using a minigene splicing experiment. For this splicing experiment, we used a previously established wild‐type minigene construct including the following components: exon 9–truncated intron 9–exon 10–truncated intron 10–exon 11 (Fukao et al. 2010) (Fig. 3A). We created the c.941‐9T>A mutant construct using in vitro mutagenesis. Since our minigene construct produces human T2‐rabbit β‐globin fusion mRNA, we could amplify the minigene‐specific mRNA by RT‐PCR using a combination of a human T2 sense primer and a rabbit β‐globin antisense primer. As shown in Fig. 3B, exon 10 skipping was induced in the case of the c.941‐9T>A mutant construct; normally spliced transcripts with the inclusion of exon 10 were also produced. In contrast, the wild‐type construct had no band corresponding to exon 10 skipping.

The above findings explain the molecular basis by which c.941‐9T>A mutation causes exon 10 skipping. c.941‐9T>A mutation at the polypyrimidine stretch of the splice acceptor site of intron 9 results in insufficient exon 10 recognition. This mutation causes exon 10 skipping in most, but not all, transcripts. The c.941‐9T>A mutation effect on splice site selection seems to be incomplete. Transcripts with exon 10 skipping are rapidly degraded by NMD. Hence, under steady‐state conditions, only normal T2 mRNA can be detected at a level <10% of that in controls. Therefore, individuals homozygous for this mutation, GK03 and GK124, had <10% activity from such residual normal T2 mRNA.

A splice acceptor site consists of two parts. One is the proximal region from −3 to +1 with the consensus sequence cag/G. The other distal region is the polypyrimidine stretch from −14 to −5. As it is usually possible for some purine residues to be present in this region, it is difficult to determine whether a nucleotide substitution in this region is a disease‐causing mutation or functionally neutral. This is one of the reasons why mutations located in polypyrimidine stretches have rarely been reported (Lewandowska 2013). In carnitine acylcarnitine translocase deficiency, c.199‐10T>G in *SLC25A20* is a common mutation, resulting in the skipping of exons 3 and 4 or exon 3 alone (Ogawa et al. 2000; Vatanavicharn et al. 2015). This mutation changed the S&S score from 92.50 (gtgattccttgcag/G) to 86.88 (gtgagtccttgcag/G). It has been reported that ag dinucleotides in polypyrimidine stretches are very rare. There are also few reports describing that single‐nucleotide substitutions in polypyrimidine stretches of splice acceptor sites were proved to cause aberrant splicing (Montejo et al. 1999; Ogawa et al. 2000; Carboni et al. 2011; Ben Rhouma et al. 2013; Lewandowska 2013; Waye et al. 2013; Mattioli et al. 2014). We thus examined whether in silico tools can predict splice aberration caused by these polypyrimidine stretch mutations (Table 1). We used Human Splicing Finder (HSF) (http://www.umd.be/HSF3/HSF.html), which combines 12 different algorithms to identify and predict the effects of mutations on splicing signals (Desmet et al. 2009). Among them, in some cases, the prediction of splicing effects was successful, but it was not in others. HSF failed to predict exon 10 skipping in the present case.

**Table 1:** Prediction of splicing effects by in silico tools

Gene	OMIM*	RefSeq	Mutation	Intron	Reference sequence	Mutant sequence	3′ Senapathy and Shapiro score		Prediction of Human Splicing Finder (HSF)		Reported effects	Ref. No.
							Reference	Mutant	HSF matrices (acceptor site)	MaxEnt (3′ motif)		
***ACAT1***	**607809**	**NM_000019.3**	**c.941‐9T>A**	**9**	ctttttttaaacag/C	ctttt**a**ttaaacag/C	**90.48**	**85.17**	**Not significant**	**Probably no impact on splicing**	**Exon 10 skipping**	**Present case**
*SLC25A20*	613698	NM_000387.5	c.199‐10T>G	2	gtgattccttgcag/G	gtga**g**tccttgcag/G	92.50	86.88	New acceptor site	Broken WT acceptor site	Exon 3 skipping or exon 3 and 4 skipping	Ogawa et al. (2000)
*LMNA*	150330	NM_170707.3	c.937‐11C>G	5	ccaccccccttcag/C	cca**g**cccccttcag/C	83.29	82.67	New acceptor site	New acceptor site or broken WT acceptor site	40‐bp insertion	Carboni et al. (2011)
*AGL*	610860	NM_000642.2	c.2682‐8A>G	20	ctctgaattttcag/G	ctctga**g**ttttcag/G	88.59	88.59	New acceptor site	Broken WT acceptor site	7‐bp insertion	Ben Rhouma et al. (2013)
*FANCA*	607139	NM_000135.2	c.710‐5T>C	7	ttatggtttttcag/A	ttatggttt**c**tcag/A	89.29	88.35	Not significant	Probably no impact on splicing	Exon 8 skipping	Mattioli et al. (2014)
*HBB*	141900	NM_000518.4	c.316‐12T>C	2	tcttcctcccacag/C	tc**c**tcctcccacag/C	89.07	86.57	Not significant	Probably no impact on splicing	Could reduce splicing efficiency	Waye et al. (2013)
*HBB*	141900	NM_000518.4	c.316‐7C>A	2	tcttcctcccacag/C	tcttcct**a**ccacag/C	89.07	88.14	Not significant	Probably no impact on splicing	Could reduce splicing efficiency	Waye et al. (2013)
*F9(FIX)*	300746	NM_000133.3	c.392‐8T>G	4	tttgcttcttttag/A	tttgct**g**cttttag/A	85.96	84.86	Not significant	Probably no impact on splicing	Exon 5 skipping	Montejo et al. (1999)
*F9(FIX)*	300746	NM_000133.3	c.392‐9T>G	4	tttgcttcttttag/A	tttgc**g**tcttttag/A	85.96	83.46	Not significant	Probably no impact on splicing	Exon 5 skipping	Lewandowska (2013)

In the mutant sequence column, mutated nucleotides are shown in bold type and underlined. WT, wild type.

We showed that c.941‐9T>A induces aberrant splicing in the *ACAT1* gene. In silico tools still have a limited ability to predict splicing abnormalities. cDNA analysis and minigene splicing experiments remain useful alternatives to reveal splice defects.

## Conflict of Interest

The authors declare no potential conflicts of interest with respect to the research, authorship, and/or publication of this article.
